# Advancements in Modern Nucleic Acid-Based Multiplex Testing Methodologies for the Diagnosis of Swine Infectious Diseases

**DOI:** 10.3390/vetsci12080693

**Published:** 2025-07-24

**Authors:** Jingneng Wang, Lei Zhou, Hanchun Yang

**Affiliations:** 1 National Key Laboratory of Veterinary Public Health Safey, College of Veterinary Medicine, China Agricultural University, Beijing 100193, China; 2 Key Laboratory of Animal Epidemiology of Ministry of Agriculture and Rural Affairs, College of Veterinary Medicine, China Agricultural University, Beijing 100193, China; 3 Shanghai Xiongtu Biotechnology Co., Ltd., Shanghai 201613, China

**Keywords:** nucleic acid-based multiplex testing, swine infectious diseases, co-infection diagnostics, multiplex qPCR, digital PCR, microarray technology, next-generation sequencing

## Abstract

Swine infectious diseases, often caused by multiple co-infecting agents, pose severe global threats to pig health and industry economics. Conventional single-plex testing assays, whether relying on pathogen antigens or nucleic acids, exhibit limited efficacy in the face of co-infection events. The modern nucleic acid-based multiplex testing (NAMT) methods demonstrate substantial strengths in the simultaneous detection of multiple pathogens involving co-infections owing to their remarkable sensitivity, exceptional specificity, high-throughput, and short turnaround time. The development, commercialization, and application of NAMT assays in swine infectious disease surveillance would be advantageous for early detection and control of pathogens at the onset of an epidemic, prior to community transmission. Such approaches not only contribute to saving the lives of pigs but also aid pig farmers in mitigating or preventing substantial economic losses resulting from infectious disease outbreaks, thereby alleviating unwanted pressure on animal and human health systems. The current literature review provides an overview of some modern NAMT methods, such as multiplex quantitative real-time PCR, multiplex digital PCR, microarrays, microfluidics, next-generation sequencing, and their applications in the diagnosis of swine infectious diseases. Furthermore, the strengths and weaknesses of these methods were discussed, as well as their future development and application trends in swine disease diagnosis.

## Simple Summary

Pigs are often infected by multiple pathogens at once, making disease diagnosis challenging and costly for farmers. Traditional tests can only detect one pathogen at a time, missing co-infections that worsen symptoms and increase mortality. This review explores advanced “multiplex” tests that simultaneously identify numerous swine pathogens in a single sample. These tests—using technologies like PCR, microchips, and genetic sequencing—are faster, more accurate, and more efficient than older methods. They help veterinarians quickly diagnose complex infections (e.g., respiratory or diarrheal diseases), enabling early intervention to save pigs and reduce economic losses. We compare the benefits and limitations of each technology and highlight their future potential in transforming swine disease management.

## 1. Introduction

Pork is widely favored as a staple meat globally. A 2022 survey indicates that pork accounts for 34% of total meat consumption worldwide [1]. With ongoing growth in global population and improving economic conditions, it is anticipated that global pork consumption will persistently rise. Concurrently, there has been a steady increase in both global pig population and production, reaching an estimated one billion pigs by 2022 [1]. In the face of such a large population, previous experiences have taught us that once infectious diseases emerge in the pig population they can lead to significant economic losses for the local or global pig industry. For instance, the widespread outbreak of African swine fever (ASF) between 2018 and 2019 resulted in approximately a 25% reduction in the global pig population [2]. Over the past five decades, infectious diseases caused by pathogens such as African swine fever virus (ASFV), foot-and-mouth disease virus (FMDV), porcine reproductive and respiratory syndrome virus (PRRSV), and porcine epidemic diarrhea virus (PEDV) have inflicted significant harm on the swine industry [3]. In the present era, the proliferation of large-scale, high-density indoor production models and the globalization of the swine industry have contributed to the dissemination of pathogens, with infectious diseases representing a primary constraint on the high-quality advancement of modern pig farming.

Many swine infectious diseases are attributed to a single pathogen in conjunction with inadequate sanitation and poor management [3,4]. However, co-infection with multiple pathogens is also prevalent in modern pig farms and clinical settings [5,6,7,8,9]. Furthermore, co-infection may occur more frequently than single infection in pig farming operations [8]. In cases of swine diseases caused by co-infection with multiple pathogens, the clinical symptoms of any single pathogen infection typically closely resemble those of other single pathogen infections or co-infections involving more than one pathogen. For example, the clinical manifestations of porcine deltacoronavirus (PDCoV) infection resemble those caused by other swine enteric pathogens such as transmissible gastroenteritis virus (TGEV), PEDV, and so forth [10]. The presence of co-infections complicates and renders more challenging the task of veterinary practitioners in identifying the primary cause of swine infectious diseases through clinical observations.

The methods employed in veterinary diagnostic laboratories for the diagnosis of swine diseases can be divided into serological and virological methods [10], of which enzyme-linked immunosorbent assay, polymerase chain reaction (PCR), and quantitative real-time PCR (qPCR) are the commonly utilized approaches for rapid detection of porcine pathogens. When pigs are co-infected with multiple pathogens, enzyme-linked immunosorbent assay or single-plex qPCR (sqPCR) cannot simultaneously detect and differentiate these pathogens in a single test run. In such scenarios, multiplex PCR (mPCR) technology offers a viable solution for the accurate diagnosis of porcine infectious diseases resulting from co-infection by multiple pathogens. The principle behind mPCR involves the simultaneous amplification of multiple target nucleic acid sequences using more than one primer sets within a single reaction [11]. The mPCR is an optimal and practical method for diagnostic purposes, enabling simultaneous identification of multiple causative agents in a single assay. Various types of mPCR techniques, including multiplex classical PCR [12,13,14], multiplex qPCR (mqPCR) [15,16,17] and multiplex digital PCR (mdPCR) [18,19,20], have been employed to develop mPCR assays for simultaneous identification of several porcine pathogens.

While mPCR technology is capable of amplifying and enriching the target sequences of multiple pathogen nucleic acids in a single test, accurate diagnostic results require the separation and visualization of the enriched target sequences using complementary detection techniques. In addition to real-time detection techniques like qPCR, various end-point detection methods—including agarose gel electrophoresis (AGE), capillary electrophoresis (CE), lateral flow dipstick (LFD), among others—are widely employed for the separation and analysis of mPCR amplicons. Consequently, numerous multiplex assays leveraging these techniques have been developed for diagnosing swine infectious diseases [14,21,22,23]. It should be noted that mPCR is not the sole technology suitable for developing multiplex diagnostic assays for swine infectious diseases. The utilization of high-throughput molecular detection techniques, such as microarrays and microfluidics, for the diagnosis of swine infectious diseases has garnered significant attention too [24,25,26,27].

The review article provides a concise overview of modern nucleic acid-based molecular detection techniques and multiplex diagnostic approaches for simultaneous detection of multiple porcine agents. Furthermore, it discusses the strengths and weaknesses of various multiplex diagnostic approaches, as well as the potential applications of these methods in swine disease diagnosis. We believe that this summary of nucleic acid-based multiplex diagnostic methods for accurate swine infectious disease diagnosis will be valuable to both veterinary practitioners and laboratory workers.

## 2. Multiplex Quantitative Real-Time PCR

As early as the 1990s, gel-based mPCR has been recognized as a specific diagnostic method for identifying bacterial and viral pathogens causing swine diseases [12,13]. While gel electrophoresis constrains the sensitivity and resolution of mPCR detection from a contemporary perspective, it nevertheless represented the standard post-amplification analysis technique for verifying PCR amplicons at the time. These constraints were not fundamentally overcome until the advent of qPCR, which enabled real-time monitoring of amplification dynamics. Compared to gel electrophoresis-based PCR detection methods, qPCR exhibits substantial improvements in sensitivity, specificity, and throughput [28]. It continues to serve as the predominant molecular diagnostic approach for human and animal infectious diseases and is recognized as the gold standard [29]. Additionally, reverse transcription is routinely integrated with qPCR to form reverse transcription-qPCR (RT-qPCR) assays for detecting swine RNA viruses [30]. Consequently, this section centers on multiplex qPCR and RT-qPCR assays for swine infectious disease diagnosis, highlighting recently developed methodologies.

### 2.1. Multiplex qPCR Assays for Swine Vesicular Diseases

Although qPCR [31,32] and mPCR [33] were initially developed in the early 1990s, the integrated mqPCR methodology for diagnosing swine infectious diseases did not emerge until the early 2000s. To our knowledge, the first mqPCR assay for swine disease diagnosis was pioneered by Rasmussen et al. in 2003 [34]. This multiplex RT-qPCR (mRT-qPCR) assay is capable of simultaneously detecting seven distinct serotypes of FMDV and providing a diagnostic result within a few hours. The limit of detection (LOD) is below the threshold equivalent to 10 genomic copies. Subsequently, a series of mqPCR assays were developed for the detection and differentiation of FMDV serotypes prevalent in Vietnam [35], India [36], Middle East [37], East Africa [38], West Eurasia [39], and Korea [40].

Another earlier report of mqPCR method is also associated with the identification of vesicular disease pathogens. In 2005, Rasmussen et al. developed an mRT-qPCR assay capable of distinguishing between the New Jersey and Indiana serotypes of vesicular stomatitis virus (VSV) [41]. The LOD for both serotypes was determined to be less than 10 TCID_50_/mL. Subsequently, majority of research reports on mRT-qPCR assay for VSV discrimination have originated from the National Centre for Foreign Animal Disease of Canada. They successively developed and validated three multiplex assays for the detection and typing of VSV in 2006 [42], 2010 [43], and 2021 [44]. Following its initial development in 2006, each subsequent enhancement was aimed at increasing the assay’s applicability to emerging strains. The recently revamped assay has significantly enhanced sensitivity in detecting the New Jersey viral lineage 2.1 [44]. In addition to FMDV and VSV, swine vesicular disease virus (SVDV), vesicular exanthema of swine virus (VESV), and Senecavirus A (SVA) can also cause clinically indistinguishable vesicular disease in pigs. Furthermore, it is challenging to make a definitive diagnosis based solely on clinical observations [45]. Consequently, researchers have developed several mqPCR assays to enable specific differential diagnosis of vesicular disease [46,47,48,49]. Notably, microarray-based multiplex assays for differential diagnosis of swine vesicular diseases have also been developed, as detailed in Section 5.

### 2.2. Multiplex qPCR Assays for Swine Reproductive Diseases

During the initial development of mqPCR, this methodology was also applied to detect and differentiate additional porcine pathogens, such as PRRSV. In 2005, an mRT-qPCR assay was developed to differentiate between European (EU) and North American (NA) PRRSV strains [50]. The results indicated that the analytical sensitivities for both viral strains were less than 0.1 TCID_50_/0.1 mL. Moreover, both strains were successfully detected even when the concentration of the two strains differed by a factor of 5000. Subsequently, multiplex tests were gradually developed for rapid differential detection of highly pathogenic (HP-PRRSV) and classical (C-PRRSV) NA strains of PRRSV [51,52]. Following the discovery of NADC30-like PRRSV (NL-PRRSV) in 2015 [53,54], subsequent research on PRRSV multiplex assays has predominantly focused on effectively differentiating HP-PRRSV, C-PRRSV, and NL-PRRSV [55,56,57,58]. The newly developed quadruplex assay achieved simultaneous differentiation of EU, C-PRRSV, HP-PRRSV, and NL-PRRSV strains, demonstrating high specificity (97.33%) and sensitivity (96.00%) [55].

Clinical manifestations of PRRSV infection including abortion, fetal mummification, and stillbirth in sows [59]—indicate reproductive impairment and are clinically similar to those induced by major swine pathogens such as classical swine fever virus (CSFV), ASFV, porcine parvovirus (PPV), and pseudorabies virus (PRV). Furthermore, co-infections involving PRRSV and one or more of these pathogens are frequently documented [9], posing challenges to the diagnosis of reproductive impairment in sows. Consequently, mqPCR technology has enabled comprehensive detection of sow reproductive disorders, establishing itself as a critical research priority. For example, a duplex RT-qPCR panel for simultaneous detection of NA-PRRSV strain and CSFV was developed and evaluated in 2008 [60]. Another SYBR Green I-based duplex assay developed in 2020 could detect PRRSV and porcine circovirus (PCV) type 3 (PCV3) [61]. Two duplex assays for differential detection of Japanese encephalitis virus (JEV) were independently developed by two distinct research teams [62,63].

Previous studies have shown a relatively higher retrieval rate of triplex and quadruplex assays for the differential diagnosis of porcine reproductive disorders. In summary, Liu et al. successively described triplex or quadruplex assays capable of simultaneously detecting and distinguishing three or four causative agents: PRRSV, CSFV, and porcine teschovirus [64], PRRSV, CSFV, and PCV type 2 (PCV2) [65], PRRSV, CSFV, and ASFV [66], classical, variant, and Bartha-K61 vaccine strains of PRV [67], PRRSV, CSFV, PCV2, and PPV [68], PCV2, PCV3, PPV and PRV [69], as well as CSFV, PRV, JEV and porcine hemagglutinating encephalomyelitis virus (PHEV) [30]. Although previous studies report multiplex assays detecting six [70,71], eight [72] or twelve [73] pathogens associated with porcine reproductive disorders, it is critical to recognize that these pathogens are not co-amplified in a single reaction. Instead, targets are processed in discrete reaction vessels, rendering these methodologies fundamentally triplex or quadruplex qPCR assays at the reaction level.

### 2.3. Multiplex qPCR Assays for Swine Respiratory Diseases

Swine respiratory diseases arise from viral and bacterial pathogens, including swine influenza virus (SIV), PCV2, PRRSV, *Mycoplasma hyopneumoniae*, and *Streptococcus suis* (*S. suis*), with comprehensive details available in references [8,74,75]. Notably, PRRSV contributes not only to reproductive disorders in sows but also to severe respiratory syndromes in piglets [76]. For concision in this review, mqPCR assays targeting PRRSV—previously detailed in the reproductive disorders section—are excluded from further discussion herein.

Porcine circovirus disease, caused by PCV2, is a prevalent respiratory illness in swine. A recent study has demonstrated that currently, at least eight PCV2 genotypes are circulating worldwide [77]. To distinguish the genotypes of PCV2, two independent mqPCR assays were developed by Gagnon et al. [78] and by Wang et al. [79], respectively, in 2008 and 2020; the former could specifically differentiate PCV2a and PCV2b, and the later could specifically identify PCV2a, PCV2b and PCV2d. With the emergence and identification of PCV3 in 2016 [80] and PCV4 in 2019 [81], numbers of mqPCR assays to distinguish PCV2, PCV3 and PCV4 have been developed successively in recent years [82,83,84,85,86,87]. Furthermore, several studies have been carried out to simultaneously detect multiple pathogens that co-infected with PCV, such as assays for simultaneous testing PCV2, PRV and ASFV [88], PCV2, PPV and CSFV [17], PCV3 and PRV [89], as well as CSFV and PCV3 [90].

SIV is a primary viral respiratory pathogen that causes respiratory tract infections in pigs [75]. Despite SIV being a primary pathogen, there are limited reports of multiplex assays associated with SIV, possibly due to its low mortality rate [91]. The most recent publication on the SIV multiplex panel was released in 2021, and its findings demonstrated that the duplex RT-qPCR assay possessed the capability to distinguish between H1N1 and H3N2 subtypes with a LOD of 5 copies/μL [92]. Several other multiplex systems for differentiating SIV subtypes have been developed and assessed by research teams in Canada [93], Germany [94], and Brazil [95,96]. Most of these tests are designed to differentiate between the porcine subtypes H1N1, H1N2, and H3N2, which are prevalent in domestic pig populations worldwide.

With the exception of causing encephalomyelitis, vomiting, and wasting disease [97], Lorbach et al. demonstrated that PHEV also induces respiratory disease in older pigs [98]. In 2019, the same research team developed a triplex RT-qPCR assay for differentiation of PHEV genotypes 1, 2, and 3 [99]. The multiplex system developed by Goto et al. may be the mRT-qPCR assay that could simultaneously detect the largest number of pathogens based on qPCR technology to date. They claimed that 16 pathogens related to swine respiratory diseases, including seven bacteria and nine viruses, could be distinguish simultaneously by the novel assay [100]. Finally, Fourour et al. developed a mqPCR assay to quantify three bacterial pathogens, achieving LODs of 14, 146, and 16 genome equivalents/μL for *Mycoplasma hyopneumoniae*, *Mycoplasma hyorhinis*, and *Mycoplasma flocculare*, respectively [101].

### 2.4. Multiplex qPCR Assays for Swine Haemorrhagic Diseases

The ASF, caused by ASFV, is widely recognized for its exceptionally high fatality rate and substantial economic impact on the swine industry. Currently, ASFV strains can be categorized into 24 genotypes according to the sequence of gene B646L [102]. To differentiate genotype 1 from genotype 2, a duplex assay targeting the ASFV E296R gene and a triplex assay targeting the ASFV B646L, F1055L and E183L genes were established by Li et al. and Qian et al., respectively [103,104]. Although no commercially available ASFV vaccine has been released yet, the MGF505R, I177L or EP402R gene-deleted ASFV strain considered to be the most promising candidate for a vaccine. Therefore, several multiplex detection assays were developed in advance to differentiate vaccine strains from wild-type strains [105,106,107].

CSFV induces hemorrhagic syndrome in swine and exhibits high transmissibility. Due to its persistent circulation in multiple regions [108], reliable diagnostics are therefore critical for effective CSF control programs. An mRT-qPCR panel for detection and genotyping of CSFV genotypes 1, 2, and 3 was developed in 2009 [109]. In addition, multiplex assay to distinguish between vaccine and wild-type strains of CSFV have also been reported, and its sensitivity for wild-type and C-strain vaccine CSFVs was 41.8 and 81.5 copies/mL, respectively [110].

Due to the highly variable clinical manifestations of ASF and classical swine fever, which may be mistaken for each other or other swine diseases such as swine erysipelas, multiplex assays have been developed to expedite the identification of the causative agent for these diseases [15,16,111,112,113]. It is noteworthy that the combination of direct PCR and mPCR can offer a rapid and reliable method for the differential diagnosis of swine diseases. Nishi et al. developed a direct-multiplex system for simultaneous detection of ASFV and CSFV by utilizing an impurity-tolerant enzyme and an anti-inhibition PCR buffer system [114]. The direct-multiplex system can utilize crude tissue homogenate directly as a PCR template, obviating the necessity for prior extraction and purification of viral nucleic acid in the test material. The test results were found to be consistent with those obtained using conventional purification methods.

### 2.5. Multiplex qPCR Assays for Swine Diarrheal Diseases

Porcine enteric coronaviruses, e.g., PEDV, PDCoV, TGEV, and porcine enteric alphacoronavirus (PEAV), are among the primary viral pathogens responsible for causing diarrheal diseases in neonatal piglets [115]. Among the four major porcine enteroviruses, PEDV-targeted multiplex assays constitute the predominant research focus, reflecting this pathogen’s high mortality rates and global dissemination. Most of the multiplex panels developed in the early stage were used to discriminate between classical and variant PEDVs [116,117,118]. With the development and application of PEDV vaccines [119], researchers have paid more attention to establish multiplex systems for differentiation of wild-type and vaccine strains of PEDV [120,121,122].

Porcine rotavirus (PoRV) and porcine bocavirus (PBoV) are two significant etiological agents of porcine diarrhea. Two mRT-qPCR assays for simultaneous detection of PoRV species were described, with one capable of diagnosing species A, B, and C successfully [123], and the other able to differentiate species A, B, C, and D simultaneously [124]. The efficacy of both assays was assessed with thousands of porcine fecal samples. In 2016, Zheng et al. developed an EvaGreen-based multiplex system for differentiating PBoV genogroups and validated it with 227 clinical samples from pigs. The sensitivity of the system was determined to be 100 copies/μL for group 1, 50 copies/μL for group 2, and 100 copies/μL for group 3, respectively [125].

It is widely recognized that a diverse array of pathogens can lead to porcine diarrhea. Furthermore, the presence of diarrhea in pigs may indicate concurrent infection with multiple diarrheal pathogens. As a result, numerous multiplex assays have been developed for the detection and differentiation of distinct porcine diarrhea pathogens. These multiplex assays can be categorized into four groups based on their ability to simultaneously amplify different pathogens in a single tube: duplex assay [126,127,128,129], triplex assay [130,131,132,133], quadruplex assay [134,135,136,137,138,139], and pentaplex assay [140]. Brief information on these multiplex assays for simultaneous differentiation of several swine diarrheal diseases pathogens was presented in Table 1.

**Table 1:** Brief information on multiplex assays for simultaneous differentiation of more than one swine diarrheal diseases pathogens.

Multiplex Type	Method	Pathogen	LOD	Infection Rate (%)	Reference
Duplex	SYBR Green I	PEDV, PCV3	34.6 copies/μL for PEDV, 61.2 copies/μL for PCV3	PEDV (43.94), PCV3 (16.67), PEDV/PCV3 (27.27)	[126]
Duplex	SYBR Green I	PDCoV, PSV	10 copies/μL for PDCoV, 100 copies/μL for PSV	PDCoV (20.2), PSV (23.2), PDCoV/PSV (13.8)	[127]
Duplex	TaqMan probe	PEDV, PDCoV	7 copies/reaction for PEDV, 14 copies/reaction for PDCoV	PEDV (52.9), PDCoV (46.4), PEDV/PDCoV (9.4)	[128]
Duplex	SYBR Green I	PEDV, PBoV3/4/5	10 copies/μL for each virus	PEDV (85.7), PBoV (46), PEDV/PBoV (28.6)	[129]
Triplex	TaqMan probe	PEDV, TGEV, PDCoV	10 copies/μL for each virus	PEDV (19.70), TGEV (0.87), PDCoV (10.17), PEDV/TGEV (3.25), PEDV/PDCoV (23.16), TGEV/PDCoV (0.22), PEDV/TGEV/PDCoV (11.90)	[130]
Triplex	TaqMan probe	PEDV, PoRV, PDCoV	60 copies/μL for each virus	PEDV (51.79), PoRV (59.82), PDCoV (2.68), PEDV/PoRV (23.21), PDCoV/PoRV (1.79%)	[131]
Triplex	TaqMan probe	TGEV, PDCoV, PEDV	5 copies/μL for TGEV, 200 copies/μL for PDCoV, 5 copies/μL for PEDV	TGEV (16.36), PDCoV (0), PEDV (78.18)	[132]
Triplex	TaqMan probe	PEDV, TGEV, PDCoV	2.95 copies/μL for each virus	PEDV (38.13), TGEV (1.88), PDCoV (5.00), PEDV/TGEV (1.25), PEDV/PDCoV (1.25), TGEV/PDCoV (0), PEDV/TGEV/PDCoV (0.63)	[133]
Quadruplex	TaqMan probe	TGEV, PEDV, PDCoV, PEAV	110 copies/reaction for each virus	TGEV (20.34), PEDV (9.60), PDCoV (34.18), PEAV (1.41), PDCoV/TGEV (3.67), PDCoV/PEDV (1.41), PEDV/TGEV (1.13), PEDV/PDCoV/TGEV (2.54), TGEV/PEDV/PDCoV/PEAV (0.56)	[134]
Quadruplex	SYBR Green I	PEDV, TGEV, PoRV, PDCoV	863 copies/μL for PEDV, 192 copies/μL for TGEV, 174 copies/μL for PoRV, 176 copies/μL for PDCoV	PEDV (19.05), TGEV (5.21), PoRV (4.32), PDCoV (3.87)	[135]
Quadruplex	TaqMan probe	PEDV, PDCoV, PToV ^a^, PEAV ^b^	100 copies/μL for each pathogen	PEDV (55.36), PDCoV (28.57), PToV (5.36), PEAV (1.79), PEDV/PToV (7.14), PEDV/PToV/PDCoV (1.79)	[136]
Quadruplex	TaqMan probe	PEDV-G1, PEDV-G2, PoRV-A, PoRV-C	20 copies/μL for PEDV-G1, 100 copies/μL for PEDV-G2, 50 copies/μL for PoRV-A and PoRV-C	PEDV-G1 (3.41), PEDV-G2 (9.09), PoRV-A (12.50), PoRV-C (11.36), PEDV-G1/PoRV-C (1.14), PEDV-G2/PoRV-A (5.68), PoRV-A/PoRV-C (3.41), PEDV-G2/PoRV-A/PoRV-C (1.14)	[137]
Quadruplex	TaqMan probe	PEDV, TGEV, PDCoV, PEAV	121 copies/μL for each virus	PEDV (18.26), TGEV (0.46), PDCoV (13.16), PEAV (0.15)	[138]
Quadruplex	TaqMan probe	PEDV, PDCoV, TGEV, PEAV	8 copies/reaction for PEDV, 4 copies/reaction for PDCoV, 16 copies/reaction for TGEV, 6.8 copies/reaction for PEAV	PEDV (72.88), PDCoV (17.58), PEDV/PDCoV (8.89), PEDV/TGEV (0.13), PEDV/PDCoV/TGEV (0.53)	[139]
Pentaplex	EvaGreen	TGEV, PEDV, PCV2, PoRV-A, PoRV-C	5 copies/μL for TGEV, 5 copies/μL for PoRV-A, 50 copies/μL for PoRV-C, 5 copies/μL for PEDV, 50 copies/μL for PCV2	TGEV (1.11), PoRV-A (1.11), PEDV (8.89), PCV2 (22.22), TGEV/PCV2 (1.11), PoRV-A/PCV2 (1.11), PoRV-C/PCV2 (1.11), PEDV /PCV2 (16.67), PoRV-C/PEDV/PCV2 (3.33)	[140]

^a^ PToV: Porcine torovirus. ^b^ As swine enteric alphacoronavirus (SeACoV), swine acute diarrhea syndrome coronavirus (SADS-CoV), and porcine enteric alphacoronavirus (PEAV) all denote the same pathogen [141], they have been uniformly referred to as PEAV in this review.

## 3. Multiplex Digital Polymerase Chain Reaction

Digital PCR (dPCR) is a novel technique for the quantification of target nucleic acids, which is based on the strategy of separately amplifying single template molecules in tens of thousands of independent sub-reactions [142]. Compared to qPCR, dPCR offers higher sensitivity, improved precision, and increased tolerance to PCR inhibitors, rendering it suitable for applications in clinical diagnosis, food and agricultural testing, as well as environmental monitoring [143]. In the diagnosis of swine diseases, dPCR has also been utilized for the detection of individual pathogens such as ASFV [144], PEDV [145], and PCV [146]. However, to date, mdPCR assays involving two or more porcine pathogens are still infrequently reported, possibly due to the limited availability of fluorescent channels on current dPCR devices.

Over the past few years, four duplex [20,147,148,149] and three triplex [18,19,150] dPCR assays were developed to simultaneously detect and differentiate more than one porcine pathogens. In these studies, the overwhelming consensus among investigators is that dPCR exhibits greater sensitivity and accuracy compared to qPCR. These advantages are particularly pronounced when dealing with a low pathogen count in clinical samples. For instance, Shi et al. illustrated that the LOD of the mdPCR assay for ASFV, CSFV, and PRRSV was tenfold higher compared to that of the mRT-qPCR assay [150]. The performance of these mdPCR assays and their comparison with qPCR assays was detailed in Table 2.

**Table 2:** Comparison of dPCR and qPCR assays based on performance.

Multiplex Type	Target	dPCR Assay			qPCR Assay			Reference
		Instrument	LOD	Positive Rate	Instrument	LOD	Positive Rate	
Duplex	glycoprotein B and E genes of PRV	QX 100 (Bio-Rad, CA, USA)	4.75 copies/μL	82.61% for tissue samples, 80.95% for serum samples	CFX96 (Bio-Rad, CA, USA)	76 copies/μL	78.26% for tissue samples, 47.62% for serum samples	[148]
Duplex	ORF2 genes of PCV2 and PCV3 ^a^	QX 200 (Bio-Rad, CA, USA)	2 copies/μL for PCV2, 1 copy/μL for PCV3	81.00% for PCV2, 8.33% for PCV3	QuantStudio 6 Flex (Applied Biosystems, CA, USA)	6 copies/μL for PCV2, 10 copies/μL for PCV3	81.00% for PCV2, 7.33% for PCV3	[147]
Duplex	ORF1ab genes of SARS-CoV-2 and PEAV ^b^	QX 200 (Bio-Rad, CA, USA)	1.48 copies/reaction for SARS-CoV-2, 1.38 copies/reaction for PEAV	100.00% for SARS-CoV-2, 18.90% for PEAV	ABI 7500 (Applied Biosystems, CA, USA)	6.18 copies/reaction for SARS-CoV-2, 14.10 copies/reaction	100.00% for SARS-CoV-2, 18.90% for PEAV	[149]
Duplex	B646L and EP402R genes of ASFV	QX 200 (Bio-Rad, CA, USA)	1 copies/μL	100.00% for B646L and EP402R	LightCycler 96 (Roche, BS, Switzerland)	10 copies/μL	95.45% for B646L, 93.18% for EP402R	[20]
Triplex	ASFV-p72, CSFV-5′ UTR and PRRSV-ORF7 ^c^	Naica (Stilla Technologies, IDF, France)	0.469 copies/μL for each virus	30.10%, 13.49%, and 22.49% for ASFV, CSFV, and PRRSV, respectively	QuantStudio 5 (Applied Biosystems, CA, USA)	4.69 copies/μL for each virus	24.57%, 8.65%, and 18.34% for ASFV, CSFV, and PRRSV, respectively	[150]
Triplex	ORF1agenes (Nsp2 region) of C-PRRSV, HP-PRRSV and NL-PRRSV	Naica (Stilla Technologies, IDF, France)	0.32 copies/μL	2.19% for C-PRRSV, 25.31% for HP-PRRSV, 11.56% for NL-PRRSV, 7.50% for co-infection	QuantStudio 5 (Applied Biosystems, CA, USA)	3.20 copies/μL	1.88% for C-PRRSV, 21.56% for HP-PRRSV, 9.69% for NL-PRRSV, 5.94% for co-infection	[19]
Triplex	B646L, MGF505-2R and I177L genes of ASFV	Naica (Stilla Technologies, IDF, France)	12 copies/reaction	14.17%	QuantStudio 6 Flex (Applied Biosystems, CA, USA)	500 copies/reaction	12.98%	[18]

^a^ ORF: Open reading frame. ^b^ SARS-CoV-2: Severe acute respiratory syndrome coronavirus 2. ^c^ UTR: Untranslated region.

## 4. Capillary Electrophoresis-Based Multiplex Detection Methods

CE is an electrically driven separation technique that uses a very thin glass capillary tube (usually with inner diameter less than 100 μm) as a separation channel under the influence of very high electrical fields [151]. Following extensive development, it has been applicable in various areas like analysis of proteins, chemical substances, metabolites, and nucleic acids [152]. Despite commercial availability of CE platforms including the Capel series (Lumex Instruments, BC, Canada), BioFocus series (Bio-Rad Laboratories, CA, USA), and PrinCE Next series (Prince Technologies, DR, Netherlands), translational adoption of CE-based multiplex assays for porcine diagnostics remains limited, with minimal peer-reviewed validation studies and clinical implementation reported to date.

The CE-based multiplex assays for the simultaneous detection of seven, eight and nine porcine pathogens were developed and validated by three independent research teams, respectively. By using the GenomeLab GeXP system (GeXP; SCIEX, MA, USA) for PCR amplification products separation and detection, Zhang et al. established an mPCR assay that detects and differentiates eight distinct pathogens (CSFV, JEV, PCV2, PRRSV-NA, PPV, PRV, SIV-H1, and SIV-H3) associated with reproductive or respiratory diseases in swine [153]. Results demonstrated that the LODs for different pathogens ranged from 10 to 1000 copies/μL. When evaluated using clinical specimens, the multiplex assay demonstrated a significantly lower detection rate for SIV-H1 (95.8%) compared to the reference qPCR method (100%). The other one GeXP-based mPCR assay was developed by Hu et al. in 2016 that could simultaneously detect seven porcine pathogens (ASFV, CSFV, JEV, PCV2, PPV, PRRSV and PRV) with a LOD of 1000 copies/μL [22]. Unlike the two GeXP-based mPCR assays described above, Wu et al. employed QIAxcel Advanced system (QIAGEN, NRW, Germany) as the PCR products detection platform and developed a CE-based mPCR assay for the simultaneous detection of nine porcine pathogens [21]. Its LOD was 1 × 10^4^ copies/μL when recombinant plasmids of the target genes of these nine pathogens were simultaneously used as templates for PCR amplification. Based on the available reports, it is evident that while the CE-based multiplex assay can simultaneously detect a greater number of pathogens, its sensitivity is notably lower compared to qPCR and dPCR multiplex assays.

## 5. Microarrays for Swine Diseases Detection and Discrimination

Microarray refers to a solid-surface support (glass slide, quartz wafer, silicon slide or microsphere, etc.) onto which thousands of discrete features are bound in an array format of microscopic spots for simultaneous analysis of multiple genetic or biochemical targets [154]. Although the microarray-based assay methods were conceived of and developed for immunoassay in the mid- to late-1980s by Ekins et al. [155], presumably the term “microarray” was first introduced in the mid-1990s by Schena et al. [156], Ferguson et al. [157] and Shalon et al. [158] for hybridization analysis of DNA samples. After decades of development, many different types of microarrays, i.e., DNA microarrays, protein microarrays and glycan microarray, have been developed and applied to various scenarios in the field of biology including gene expression analysis, determination of protein/glycan–protein interactions and diagnosis of infectious diseases [159]. Due to the advantages of high-throughput sample screening, simultaneous detection of multiple targets and small quantity of sample requirements, microarray technology has been widely applied in the identification and discrimination of porcine pathogens. This section focuses on the research of DNA microarrays for detecting porcine pathogens. DNA microarrays employed in these studies are classified as two-dimensional solid arrays or three-dimensional suspension bead arrays, with concise introduction and discussion provided for each format.

### 5.1. Two-Dimensional Solid Arrays

In 2006, an oligonucleotide microarray for detection and typing of all seven FMDV serotypes (A, O, C, Asia 1, SAT1, SAT2 and SAT3) [160] and a cDNA microarray for simultaneous detection of FMDV and PRRSV [161] were developed. These may represent some of the earliest DNA microarrays specifically designed for pathogen detection in pigs. Subsequently, a series of microarrays for detection and characterization of pathogens causing vesicular or vesicular-like symptoms in swine have been developed by different research communities [162,163,164]. Among the earliest DNA microarrays for detecting bacterial diseases in swine, Han et al. developed a microarray targeting Escherichia coli virulence factors [165]. This platform simultaneously identified O-antigen-specific genes of all eight major *Escherichia coli* (*E. coli*) serogroups and 11 genes encoding exotoxins and adhesins implicated in post-weaning diarrhea and edema disease. For the discrimination of PCV genotypes, two oligonucleotide microarrays were successively released in 2008 [166] and 2010 [167], both of them were capable of unambiguous distinguish of PCV1 and PCV2. A key distinction between the two PCV genotyping microarrays is that results from the microarray developed by An et al. can be visually assessed with the naked eye, eliminating the need for specialized instruments [166].

Admittedly, more DNA microarrays have been developed for simultaneous detection and identification of multiple porcine pathogens and subtypes [24,25,168,169,170,171,172] including simultaneous testing and typing of four [24,168], six [169,172], seven [25,170] or eight [171] targets. For instance, the magnetic bead microarray described by LeBlanc et al. detected CSFV, border disease virus (BDV) and bovine viral diarrhea virus (BVDV), and further differentiated between type 1 and type 2 BVDV [168]; the automated electronic microarray developed by Lung and colleagues could simultaneously detect and differentiate eight viruses and bacteria commonly associated with porcine respiratory disease complex [171]. It is worth mentioning that among these microarrays, the electronic microarray assays that utilize electric fields to control nucleic acid transport for active hybridization [25,171] are markedly different from others that rely on passive transport for nucleic acid hybridization. The greatest feature of an electronic microarray is that it automates and integrates all post-PCR steps required for microarray analysis, with the consequent benefit of greatly reducing the time, labor, and number of equipment required to obtain microarray results.

Beyond custom microarrays targeting specific porcine pathogens, two studies have evaluated the applicability of commercial broad-spectrum DNA microarrays for detecting porcine pathogens. Nicholson et al. evaluated the utility of the Virochip platform to detect swine viruses, and the results demonstrate that it could successfully detect PRRSV, PCV2, influenza A virus and porcine respiratory coronavirus in serum, lung lavage fluid or turbinate tissue homogenate of pigs [173]. Another study investigated the practicality and sensitivity of a pathogen microarray (Lawrence Livermore Microbial Detection Array) in clinical specimens from pigs, which was designed to detect 8101 microbial species [174]. This microarray could easily identify PCV2 and PRRSV, as well as several bacteria linked with PRRSV infection, but its sensitivity is about two orders of magnitude less than that of qPCR detection methods.

### 5.2. Three-Dimensional Suspension Bead Arrays

Suspension-bead-based arrays are distinct from two-dimensional solid arrays, they are essentially three-dimensional arrays which employ fluorescent encoding microspheres as solid support and flow cytometry for targets and microspheres detection [175]. In contrast to planar arrays, the three-dimensional suspension bead arrays offer many significant advantages such as faster binding kinetics and higher quality of results [176]. Currently, several commercially available suspension array platforms have been released, e.g., Luminex 100/200/FLEXMAP 3D System (Luminex Corporation, TX, USA), Bio-Plex 200/3D System (Bio-Rad, CA, USA), a variety of solution-phase multiplex assays for swine diseases detection have been developed based on these suspension array platforms.

Two oligonucleotide suspension microarray assays for differential diagnosis of four pestivirus species, namely CSFV, BDV, BVDV1 and BVDV2, that infect domestic pigs, wild boars, and other livestock, have been developed previously, one of which combined asymmetric PCR with Luminex suspension bead technology [177], the other one demonstrated the utility of combining qPCR with Luminex suspension bead system [178], and both of them found to be highly specific and sensitive. To differentiate FMDV from other viruses that cause clinically similar vesicular diseases, two multiplexed suspension microsphere assays have been reported in 2008 [179,180]. Both assays could simultaneously screen for seven DNA and RNA viruses in a single tube by using mPCR amplification coupled with Luminex xMAP technology.

*S. suis* is an important swine pathogen characterized by significant serotype diversity, with 35 distinct serotypes (1–34 and 1/2) identified based on capsular polysaccharide antigens [181]. With the advantage of microarray technology to detect multiple targets at the same time, a 32-plex [182], an 18-plex [183] and a 6-plex [184] *S. suis* serotyping assays were constructed for simultaneous identification of 33, 17 and 4 serotypes, respectively. In addition, two studies about suspension microarray for the genotyping of specific swine viruses were retrieved from previous reports. The genotyping array developed by LeBlanc et al. was able to discriminate 22 genotypes of ASFV [185]. Wang et al. developed a Luminex xTAG-based assay that could detect type 2 PRRSV and distinguish the field strains from four vaccine strains used in the USA [186].

Several suspension bead arrays have been previously described for simultaneous detection of multiple pathogens responsible for swine respiratory and reproductive diseases, as well as swine diarrheal diseases. An asymmetric mPCR-Luminex xMAP technology-based assay for simultaneous detection of CSFV, PCV2, PPV, PRRSV and PRV was established in 2015 [187]. The LOD of this assay ranged from 2.2 (PCV2) to 22 (PRRSV) copies/μL when recombinant plasmids were used as amplification templates. Another multiplex assay for the detection of seven respiratory and reproductive viruses was constructed by Xiao et al. [188]. Recently, Shi et al. established a multiplex assay for simultaneous detection of 11 diarrheal viruses including BVDV, PEDV, PTV, TGEV, PDCoV, PSV, PoRV, PToV, porcine kobuvirus (PKoV), porcine astrovirus (PAstV), and porcine sapovirus. Furthermore, this assay was employed to examine the incidence of diarrheal viruses in pig farms across Shanghai. The investigate results showed that PKoV was the most frequently detected virus in diarrheal pigs with a rate of 38.65%, followed by PAstV (20.32%) and PEDV (15.54%) [189].

## 6. Microfluidics-Based Multiplex Detection Methods

Microfluidics is the manipulation or processing of an extremely small amount of fluid (nanoliters or picoliters) through interconnected networks of channels with dimensions of tens to hundreds of micrometers, also known as lab-on-a-chip [190]. Since its introduction in the early 1990s [191], microfluidics has become one of the most promising technologies and successful applications in diagnosis, therapeutics and food and consumer safety due to its ability to consume small quantities of reagents, short times for analysis and relatively low cost [190,192]. Furthermore, a considerable advantage of the microfluidics is that it can perform the necessary steps of nucleic acid detection such as nucleic acid extraction, PCR amplification and signal detection on a single chip, and this process can be highly automated [27]. Although microfluidics has many advantages, and there are many examples of successful application in biomedical diagnostics [192], to date, the application of microfluidic technology for simultaneous identification of multiple porcine pathogens is still limited, especially when the nucleic acid of pathogen is used as the detection target.

Here, some specific cases of microfluidics-based assays for simultaneous detection of multiple porcine pathogens are briefly summarized. Most of them were developed with commercially available microfluidics platforms [26,27,193,194,195,196,197]. For instance, the high-throughput microfluidic detection systems for simultaneous identification of 18 bacterial and viral pathogens [194] or subtyping of SIV [193] both constructed on the basis of the 48.48 dynamic array chip and BioMark system (Fluidigm Corporation, CA, USA). When using the BioMark system, a pre-amplification of the extracted nucleic acid is required prior to high-throughput qPCR reaction performed on the BioMark platform. On the one hand, the additional pre-amplification step lowers the Ct values and improves the sensitivities of the BioMark system-based microfluidic assays [193,194]. However, on the other hand, it may make the detection process more complicated and laborious and increase the risk of PCR contamination. Centrifugal microfluidics is another type of commercial microfluidics platform that has been commonly used in combination with loop-mediated isothermal amplification (LAMP) for simultaneous detection of multiple porcine pathogens on a single CD-like chip [26,195,196].

In addition, a small number of in-house developed non-commercial microfluidic devices have also been used for differential diagnosis of pig diseases, e.g., the 3D-printed microfluidic device [198], the handheld visual LAMP microfluidic chip [199], and the Hive-Chip-direct LAMP multiplex and visual detection platform [200]. However, among all the reports mentioned above, only the fully integrated and automated microfluidic multiplex assay developed by Lung et al. could truly accomplish the objective of sample-to-answer [27]. Table 3 lists the microfluidics-based multiplex assays that have been applied for simultaneous identification of multiple porcine pathogens.

**Table 3:** Microfluidics-based multiplex assays reported in the literature.

Readout Method	Microfluidic Chip Type	PCR Type	Dye for Detection	Volume of the Reaction Chamber	Force of the Solution Flow	Degree of Automation	Target	Analytical Sensitivity	Reference
Encompass Optimum workstation	Microfluidic CARD	Reverse transcription PCR	Biotin	Unknown	Pressure provided bypneumatic pump	Fully automatic	ASFV, CSFV, FMDV, SVDV, and VSV	20.0–7.06 × 10^4^ TCID_50_/mL	[27]
BioMark system	BioMark dynamic array 48.48	RT-qPCR	TaqMan probe	About 3 nanoliter	Pressure provided bypneumatic pump	Unintegrated nucleic acid extraction, and required pre-amplification	Subtyping of the H1, H3, N1, and N2 lineages of SIV	2–5 log_10_ ^a^	[193]
BioMark system	BioMark dynamic array 48.48	RT-qPCR	TaqMan probe	About 3 nanoliter	Pressure provided bypneumatic pump	Unintegrated nucleic acid extraction, and required pre-amplification	Porcine cytomegalovirus, PCV2, PCV3, PRRSV-EU, PRRSV-NA, Rotavirus A, SIV, *B. pilosicoli*, *L. intracellularis*, *E. coli* type F4, *E. coli* type F18, *M. hyopneumoniae*, *A. pleuropneumoniae*, *P. multocida*, *S. suis 2*, *B. bronchiseptica* and, *M. hyorhinis*	10^−3^–10^−8 b^	[194]
RTisochip-B	CD-like chip	LAMP	Calcein	1.4 μL	Centrifugal force	Unintegrated nucleic acid extraction	FMDV, CSFV, PRRSV-NA, PCV2, PRV, and PPV	3.2 × 10^2^ copies/reaction	[195]
AJYGeneTec TM MA2000	CD-like chip	LAMP	SYBR Green	5 μL	Centrifugal force	Unintegrated nucleic acid extraction	PDCoV, PEDV, AND PEAV	10 copies/μL for PEDV, 100 copies/μL for PEAV and PDCoV	[196]
In-house developed CCD camera	3D-printed chip with four reaction chambers	LAMP	EvaGreen	20 ± 1 μL	Capillary force	Unintegrated nucleic acid extraction	PEDV, PDCoV, and TGEV	10 copies/reaction for PEDV and PDCoV, 100 copies/reaction for TGEV	[198]
SWA-01	In-house developed chip with eight reaction chambers	RT-qPCR	SYBR Green	2 μL	Unknown	Unintegrated nucleic acid extraction	PCV2, PRRSV, PEDV, and PRV	1 copy/μL for PCV2, 10 copies/μL for PRRSV and PEDV, 100 copies/μL for PRV	[197]
Naked eye (Colorimetry)	In-house developed fan-shaped microfluidic chip	LAMP	WarmStart^®^ Colorimetric Master Mix	About 10 μL	Centrifugal force	Unintegrated nucleic acid extraction	PEDV, TGEV, PoRV, and PCV2	100 copies/μL	[199]
AJYGeneTec TM MA2000	CD-like chip	LAMP	SYBR Green	5 μL	Centrifugal force	Unintegrated nucleic acid extraction	ASFV, PRV, PPV, PRRSV and, PCV2	10–100 copies/μL	[26]
UV-light device (365 nm)	Hive-Chip	LAMP	Calcein	25 μL	Capillary force	Unintegrated nucleic acid extraction	*B646L*, *B962L*, *C717R*, *D1133L*, and *G1340L* genes of ASFV	30–50 copies/μL	[200]

^a^ Comparisons of the Cq-values for the dilutions of virus isolates indicate that the microfluidic assay has a dynamic range of 2–5 log_10_, while the typical qPCR system exhibits a dynamic range of 4–6 log_10_. ^b^ Analytical sensitivity of the microfluidic assay ranges from 10^−3^ to 10^−8^, and it is identical or has 1 log_10_ difference between the microfluidic assay and the typical qPCR system.

## 7. Isothermal Amplification-Based Multiplex Detection Methods

Isothermal amplification (IA), an in vitro nucleic acid replication technique, predates PCR by two decades (invented in 1965) [201], yet its widespread adoption and application are significantly less than that of PCR. In the post-PCR era, the first published IA method was self-sustained sequence replication, followed by 23 IA methods with different amplification methods and features such as nucleic acid sequence-based amplification, recombinase polymerase amplification (RPA) and LAMP [202]. IA methods provide ideal candidates for point-of-care (POC) testing with the outstanding features of reducing duration times, simplifying instrumentation, and increasing accessibility, especially in remote rural areas or resource-limited scenarios [203]. However, not all IA methods find regular applications in the field of veterinary diagnostics. For the diagnosis of swine diseases, IA methods are usually incorporated with microfluidics, LFD, clustered regularly interspaced short palindromic repeats (CRISPR) and CRISPR-associated (Cas) proteins (CRISPR-Cas system), fluorescent probe, and AGE technologies to detect multiple porcine pathogens simultaneously. Because the microfluidics-IA-based multiplex assays have been described in Section 6 of this review [26,195,196,198,200], they will not be repeated. However, an overview of the remaining IA-based multiplex detection methods is presented in this section.

In 2018, Wang et al. developed an RPA-fluorescent probe-based duplex assay for differentiation of wild-type and gE-deleted PRV [204]. The fluorescent probes were also used as readout methods in a duplex insulated isothermal PCR (iiPCR) assay that could rapidly detect and differentiate genotypes 1 and 2 of ASFV [205] and a dual enzymatic recombinase amplification assay for identification PEDV and PoRV-A [206]. For the iiPCR assay, the PCR amplification and fluorescence signal acquisition were carried on a commercial handheld nucleic acid analyzer. The test results for positive and negative samples were automatically displayed on screen as “+” and “−”, respectively. Two LAMP-LFD multiplex assays were described, one of which for simultaneous detection of PCV2 and PEDV [207] and the other could distinguish PEDV, PoRV, and PBoV [23]. The results of testing clinical samples indicated that the diagnostic sensitivities and specificities of these LAMP-LFD assays were highly consistent with the qPCR assays. In addition, a duplex RT-RPA assay developed with RPA-AGE technology for simultaneous detection of PEDV and PDCoV in pigs has also been reported [208].

CRISPR-Cas system is one of the important transformative forces in life sciences today. Since it was first utilized as a molecular diagnostic tool in 2016 [209], large numbers of diagnostic systems related to the CRISPR-Cas system for both human and animal diseases have been continuously emerging [210,211]. Recently, the IA coupled with CRISPR-Cas system has also been applied to simultaneously detect multiple porcine pathogens or differentiate variants of virus [212,213]. For instance, the RPA-CRISPR-Cas12b/Cas13a assay for discriminating between MGF505-2R gene deletion and highly lethal wild-type ASFVs developed by Wang and colleagues [213]. The test results can be visualized through LFD or fluorescence colorimetric method with LODs of 8 copies/μL for both read out methods.

## 8. Next-Generation Sequencing as a Multiplex Diagnostic Method for Swine Diseases

Next-generation sequencing (NGS), also termed massively parallel sequencing or high-throughput nucleotide sequencing, refers to a genre of nucleotide sequence analysis techniques that enables independent and simultaneous analysis of thousands to billions of nucleotide sequences [214], which is different from the traditional Sanger sequencing. Since the first commercial NGS sequencer was launched by 454 Life Sciences (acquired by Roche) in 2005 [215], a number of different NGS platforms have become available today. Nowadays, the commonly used NGS platforms include MiSeq (Illumina, USA), Ion Torrent (Thermo Fisher Scientific, USA), Revio (PacBio, USA), MinION (Oxford Nanopore Technologies, United Kingdom), DNBSEQ (MGI Tech, China), QNome (Qitan Tech, China). Benefiting from the significant advancements of NGS platforms in terms of overall performance and cost efficiency, NGS technology has become a routine means for tumor marker or pathogen detection in the human medical field [214,216]. Although NGS technology has been routinely used in human medicine, it is still primarily employed for research-related applications in veterinary medicine [217].

To explore the infectious agents of postweaning multisystemic wasting syndrome (PMWS), the random multiple displacement amplification and NGS method has been used to investigate the virome of lymph nodes from pigs suffering from PMWS [218,219]. The results demonstrated that most of the gene sequences belonged to PCV2, but the genome sequences of PBoV, Torque Teno virus, PPV, etc., were also found. Furthermore, co-infection with more than three viruses was common in pigs suffering from PMWS and healthy pigs. More studies have applied NGS approaches to analyze fecal viral communities in diarrheal piglets or grower-finisher pigs [220,221,222,223,224,225]. These revolutionary diagnostic tools enable simultaneous detection of multiple RNA and DNA viruses in the feces of diarrheic pigs and provide a better perspective to monitor for circulating porcine viruses in pig farms.

Metagenomic NGS (mNGS) is one of the commonly used NGS technologies for detection of porcine pathogens. Because of the inherent unspecific nature of mNGS and the interferences of host genes and non-pathogenic nucleic acids, the overall sensitivity of mNGS is usually lower than that of qPCR [226,227]. Therefore, to increase the sensitivity of pathogen detection, the pre-lysis and post-lysis enrichment strategies have been described. As a result, targeted sequence capture could effectively increase sensitivity and sequence depth [228], while targeted depletion of host DNA could decrease the data volume required for sequencing analysis [229]. Chen and coworkers for the first time accurately measured the sensitivity of the nanopore-based mNGS protocol for the identification of swine pathogens and displayed it with a LOD, and the LODs for PEDV and PRRSV were 9.0 × 10^4^ and 2.3 × 10^2^ copies/reaction, respectively [227]. As a typical representative of the third-generation sequencing technology, nanopore-based DNA sequencing has the characteristics of real-time observation of sequencing results and portability, which can be used as a POC diagnostic. It has been successfully applied for the clinical detection of porcine pathogens [227,229] and the control of transboundary animal diseases (ASFV, CSFV and FMDV) [230].

## 9. Strengths and Weaknesses of Different Multiplex Diagnostic Methods

The primary issue that needs to be addressed for any diagnostic method is whether it is fit for purpose. Veterinary practitioners have been seeking a diagnostic method that possesses the unique characteristics of high specificity, high sensitivity, cost-effectiveness, and user-friendly control, while also being capable of rapidly providing accurate and reliable diagnostic results to meet the diverse requirements of different application scenarios. However, to date, no single diagnostic test can fully meet all necessary requirements [231]. Compared to conventional PCR or sqPCR, the distinguishing and noteworthy characteristic shared by various nucleic acid-based multiplex diagnostic methods is their exceptional capacity to simultaneously screen multiple pathogens from a single test sample. However, different nucleic acid detection technologies possess distinct inherent technical characteristics, thus necessitating that the multiple diagnostic methods developed based on them also have their own respective advantages and disadvantages (Table 4).

Among the multiplex diagnostic methods discussed, mqPCR is widely adopted for swine disease diagnosis owing to its high sensitivity, rapid processing time, and broad applicability relative to alternative techniques [232]. However, the ability of mqPCR to simultaneously detect multiple targets is constrained by the limited number of fluorescent channels in current commercial real-time thermal cyclers [233]. The mdPCR method suffers from a similar limitation to mqPCR [143,233], although its sensitivity is more than a dozen times higher than that of mqPCR assay [150]. By contrast, microarrays can simultaneously identify up to thousands of analytes but with relatively low sensitivity [174], even more so for oligonucleotide microarray and cDNA microarray [160,161]. However, if the specific pathogen targets are amplified by a mqPCR prior to microarray analysis, the sensitivity of the mqPCR-microarray multiplex assay can be greatly improved [187].

Among the considerable advantages of IA technology are its constant and relatively low amplification temperature, short amplification time and high tolerance to inhibitors. The significant benefits of microfluidics for diagnostic applications are its ability to integrate all testing steps on one chip and to perform different diagnostic tests within one device, as well as its low consumption of test samples and reagents. Therefore, combining microfluidics with IA technology to simultaneously detect multiple porcine pathogens in a portable, economical and rapid way provides a good opportunity to develop novel POC diagnostic tools [234]. However, some inherent defects of IA technology, such as a tendency to generate artifacts and high risk of contamination, hinder the rapid development of this new multiple POC diagnostic method.

The co-application of mPCR and CE technology, mPCR for its ability to amplify multiple targets simultaneously in a single PCR tube [235] and CE for its capacity to separate lots of amplicons simultaneously in one run with high resolution [151], theoretically results in a reliable and powerful diagnostic method for the diagnosis of pig diseases. In contrast, the LODs of current mPCR-CE-based multiplex assays significantly exceed those of widely used qPCR or mqPCR tests [21,22,153]. Since many commercial NGS platforms with different kinds of brands, types and features are available now, it is difficult to summarize their advantages and disadvantages in the application of swine diseases diagnosis with one sentence. In brief, a major advantage of NGS is unbiased sequencing, which enables identification and characterization of known, unknown and even unexpected microorganisms in diseased pigs [222,225]. However, the NGS is usually plagued by issues of huge initial capital investment, high cost per experiment and time-consuming. Therefore, NGS has not been established as a routine method for swine disease diagnosis currently, primarily due to its high cost, technical complexity, and limited accessibility in field settings.

**Table 4:** Comparison of nucleic acid-based multiplex diagnostic methods for diagnosing swine diseases.

Diagnostic Tests	Sensitivities	Specificities	Maximum Number of Targets	Maximum Number of Samples	Total Turnaround Times ^a^	Main Strengths	Main Weaknesses
Multiplex qPCR ^b^	High (most of the mqPCR-based assays have LOD at 10–100 copies/μL)	High	Up to 6 for probe-based method Up to 10+ for melting curve analysis-based method	384	2–3 h	Real-time detection, no post-PCR manipulation required High sensitivity, specificity and accuracy Wide dynamic range Low contamination risk	Limited multiplex capability Prone to non-target nucleic acids and inhibitors
Multiplex dPCR	Approximately 10-fold higher than mqPCR	High	Usually less than 7 for optical channels-based only Up to 10 for amplitude-based method Dozens of for fluorescence encoding technology ^c^	96	2–9 h	Higher sensitivity and precise Higher tolerance for inhibitors Less affected by poor amplification efficiency	Limited multiplex capability High contamination risk Narrow dynamic range Lower sample throughput per run More complicated testing procedure Large initial capital investment for instrument More expensive reagents and consumables
CE-based multiplex diagnosis	High ^d^	Higher than mqPCR	Hundreds	384	2–3 h	High sensitivity and accuracy Higher specificity More conducive to design mPCR amplification system Separation and detection of targets with high resolution and efficiency from two dimensions by using fluorescent markers and amplicon size	More complicated testing procedure Large initial capital investment for instrument Higher contamination risk
Microarrays	Generally lower than mqPCR	High	Thousands	384	From a few hours to a few days depending on different microarray platforms	Ability to simultaneously identify large numbers of analytes Smaller quantity of sample requirements High-throughput sample screening	Required more manipulations (e.g., hybridization, washing, and even additional PCR amplification) Required more skilled laboratorians Huge initial capital investment for instrument Higher contamination risk Difficult to compare quantitative data Difficult to interpret and manage test results
Microfluidics	High	It depends on the nucleic acid amplification technology combined with microfluidics	Thousands	Thousands	2–6 h	Rapid to perform and portability Low consumption of reagents Less inherent human error Safe and quick disposal of bio-hazardous wastes	Lack of affordable, simple to process materials for large-scale production of microfluidic chips Lack of programmable microfluidic chips Difficulty of sample preparation Difficulty of integrating separate microfluidic chips
IA-based multiplex diagnosis	High	Lower than mqPCR	It depends on the detection technique combined with IA	It depends on the detection technique combined with IA	Usually less than 2 h	Increased tolerance to PCR inhibitors Required less amplification times and sample preparation steps Higher amplification efficiency	More complex primer design Prone to non-specific amplification and false positives Required more enzymes and reaction components
NGS	Generally lower than mqPCR	High	Hundreds	96	From a few hours to more than 10 days depending on different sequencing platforms	Unbiased identification of known and unknown pathogens Ability to obtain complete pathogen genetic information Ability to simultaneously identify large numbers of pathogens	Huge initial capital investment for instrument Required highly skilled laboratorians and bioinformaticians Long turnaround times Difficulty of interpreting test results High Data storage costs

^a^ Herein, the term “Total turnaround times” denotes the duration from nucleic acid extraction to obtaining a test result. We have arbitrarily set the time required for nucleic acid extraction at one hour. ^b^ Currently, mqPCR-based assays are widely utilized for the diagnosis of swine diseases due to their high sensitivity, specificity, and accuracy compared to traditional serological methods. Therefore, the mqPCR-based method serves as a reference point in this study, against which other types of multiplex diagnostic methods are evaluated in terms of specificity and sensitivity. ^c^ The number of targets for fluorescence encoding technology can be determined using the formula 2^N^ − 1, where N represents the number of fluorescence channels in the dPCR instrument (http://www.turtle-tech.com.cn/technology-en/, accessed on 19 May 2025). ^d^ Although the sensitivity of the CE-based multiplex assays for swine diseases diagnosis described in this review article is not as robust as that of mqPCR assays, with a LOD ranging from 1 × 10^1^ to 1 × 10^4^ copies/μL [21,22,153], research in other domains has indicated that mPCR-CE technology can achieve comparable sensitivity to qPCR technology [236].

## 10. Conclusions and Future Perspectives

### 10.1. Implications from Human Disease Multiplexed Diagnostic Methods

Although lots of nucleic acid-based multiplex testing (NAMT) methods have been described for diagnosis of swine infectious diseases, few have been transformed into commercially available products and applied in routine swine disease diagnosis. In contrast, NAMT has taken tremendous leaps in scientific research and technology translation within human diagnostic medicine [237]. Currently, numerous commercial NAMT platforms and assays are available, such as the BioCode^®^ MDx-3000 System and BioCode^®^ Gastrointestinal Pathogen Panel (Applied BioCode, Inc., USA), as well as the MAGPIX^®^ System and NxTAG^®^ Respiratory Pathogen Panel v2 (Luminex Corporation, USA). While NAMT technology is primarily developed to meet the market demands of human medicine, it is important to note that there are no technical or methodological limitations between human and veterinary medicine in the development of multiplex diagnostic assays with NAMT technology. Hence, it is imperative to investigate the reasons behind the delayed progress in research, translation, and implementation of NAMT in veterinary medicine compared to human medicine. We propose that there are at least three significant impediments constraining the rapid advancement of NAMT in veterinary medicine.

Firstly, technological innovation and product commercialization are typically driven by market demands. Market-driven technological innovation and product commercialization prioritize human disease diagnosis, where demand substantially exceeds that for animal disease applications. Therefore, scientific research personnel, product manufacturers, and commercial investors are more inclined to engage in technological innovation, investment, as well as product development and commercialization in human medicine. It is only when the new technology has reached a relatively mature stage of development or is close to being perfected in human medicine that it will gradually be extended and expanded to the field of veterinary medicine.

Secondly, cost and cost-effectiveness are key considerations among consumers within the veterinary industry, particularly those residing in low- and middle-income countries [238]. Typically, animal health analysis products are priced 50% to 70% lower than equivalent human health analysis items [232], influencing the adoption of new animal disease testing solutions by farmers and livestock owners. Essentially, these stakeholders prefer widely used testing products that fulfill their basic daily requirements at competitive prices. Conversely, there is limited interest in newly developed testing solutions leveraging emerging technologies despite offering enhanced performance or user convenience due to their higher pricing. Moreover, if significant one-time investments are required for acquiring instruments necessary for routine usage of such innovative solutions by farmers and livestock owners, their acceptance may diminish further. In this scenario, the impetus for product manufacturers to develop and produce commercialized diagnostic solutions for veterinary users would be insufficient while the investment motivation of business investors would also significantly decrease.

Thirdly, in comparison to the field of human medicine, there appears to be a greater scarcity of qualified practitioners capable of adeptly mastering and applying advanced testing methods within the veterinary domain. The operational procedures and testing processes for emerging methods such as dPCR, microarray, and NGS are notably more intricate, presenting a substantial challenge for veterinary practitioners. The severe shortage of skilled labor and professional veterinary inspectors in the swine industry should not be unique to developing countries like Latin America and Africa [2,239]; other nations may also encounter similar challenges.

While the advancement of NAMT in swine disease diagnosis may not parallel that in human disease diagnosis, it is noteworthy that the current state of NAMT in human medicine serves as a valuable reference for the future development of multiplex diagnostic assays for swine diseases. It is anticipated that NAMT methods will be widely and extensively applied to swine disease diagnosis in the future, akin to their widespread use in human medicine.

This expectation is primarily based on (i) the diversity of pathogens in porcine infectious diseases and the occurrences of concurrent infections by multiple pathogens in swine are increasingly prevalent. During co-infection events, the NAMT methods possess a unique advantage in detecting, identifying, and differentiating pathogens and/or pathogen genotypes; (ii) As global swine husbandry continues its expansion and large-scale industrialized pig farms play an increasingly dominant role in the industry [1], there will be a rising demand for NAMT diagnostic assays tailored specifically for pig diseases within the market. Because large-scale pig farmers are anticipated to exhibit a greater propensity for adopting new technologies and have a higher capacity to absorb the costs of new products compared to small-scale pig farmers; (iii) In the context of swine disease diagnosis, there is no longer a need to be troubled by the substantial investment required for developing NAMT technology and resolving the various challenges encountered during its initial development and application. The widespread adoption and utilization of NAMT methods in human disease diagnosis present a strong advantage for latecomers.

Therefore, by fully harnessing the potential of NAMT technology and thoroughly exploring the genuine needs of pig farmers for pig disease diagnostic products, as well as strengthening communication between diagnostic product producers and end-users, NAMT assays with the capability to simultaneously detect multiple swine diseases, while possessing high sensitivity, good specificity, and short turnaround time, are expected to garner increasing favor among farmers and pig breeders.

### 10.2. Advantages and Challenges of Nucleic Acid-Based Multiplex Testing Methods

While each NAMT method has its own distinct strengths and weaknesses (Table 4), collectively they demonstrate numerous shared advantages. The shared advantages are (i) the primary advantage of the NAMT method lies in its capability to concurrently detect and identify multiple pathogens from a single patient sample. In scenarios where pigs are infected with multiple pathogens that can induce similar clinical symptoms, and veterinarians encounter challenges in making an accurate diagnosis based on observing manifestations, this advantage will provide a valuable assistance to promptly establish a conclusive diagnosis and implement timely treatment or preventive measures; (ii) The turnaround time of 2 to 6 h from nucleic acid extraction to the final delivery of diagnostic conclusions for most nucleic acid multiplex diagnostic assays underscores the overall efficiency of the NAMT approach. This is particularly crucial in mitigating the spread of infectious swine diseases, necessitating prompt identification, isolation and culling of positive cases; (iii) The high-throughput screening of 96 to hundreds of samples at one run holds critical significance, particularly during a pandemic when diagnostic centers are overwhelmed by a substantial number of patient samples; (iv) Most NAMT methods exhibit exceptionally high sensitivity and specificity, enabling the precise detection of target pathogens at minimal pathogen loads amidst a substantial presence of non-pathogenic microorganisms. This capability facilitates early diagnosis of pig diseases and ultimately contributes to the prevention of widespread transmission of infectious diseases among pigs; (v) The NAMT method offers a cost-effective approach, enabling pig farmers to efficiently screen their entire herd for multiple infectious diseases while minimizing economic, temporal, and labor expenditures. Per single-test analysis, NAMT methodology usually incurs higher costs than conventional sqPCR assays. Despite both methods involving a single test, the amount of pathogen-related information generated by the NAMT method may be tens or hundreds of times greater than that obtained from the sqPCR method. The sqPCR method would necessitate repeated testing on the same sample multiple times to achieve an equivalent level of pathogen information as a single test using the NAMT method. In this scenario, the cost of using the sqPCR method would not only increase significantly but could even surpass that of the NAMT method. Additionally, expenses associated with time and labor for conducting multiple tests on a single sample using the sqPCR method are expected to escalate significantly.

Despite the numerous advantages of the NAMT method, it is imperative to acknowledge the inherent difficulties and challenges encountered in its application for pig disease diagnosis. To date, research on the development of multiplex diagnostic assays for swine diseases using the NAMT platform has primarily been driven by academic institutions. One of the key current challenges for NAMT is how to expedite the translation of academic research findings into end-user products and facilitate rapid comprehension among end-users regarding the advantages offered by NAMT, thereby fostering their willingness to embrace these novel technologies and products. At present, the prices of analytical instruments such as dPCR machines, NGS platforms and microarray analysis platforms, which are capable of simultaneously analyzing numerous nucleic acid targets, are generally high. This poses a significant financial challenge for most pig farmers. Therefore, addressing the issue of reducing the cost of these analytical instruments to alleviate the financial burden on end-users represents another formidable obstacle for NAMT.

The most significant challenge that NAMT will inevitably encounter in its application to diagnosing swine diseases lies in the analysis and interpretation of detection results. The capacity to acquire a substantial volume of pathogen-related data for disease diagnosis in a single assay represents the primary advantage that NAMT offers for diagnosing infectious diseases in pigs. However, it also introduces novel issues and challenges to the field of porcine infectious disease diagnosis. When the NAMT method detects multiple pathogens simultaneously in a biological sample from a clinically suspect pig, novel challenges emerge that are not encountered when sqPCR is employed. For instance, determining the primary pathogens responsible for swine diseases, establishing the temporal sequence of pathogen manifestation during disease progression, assessing stage-specific impacts of pathogens on host health, and analyzing inter-pathogen interactions and pathogen-swine interaction mechanisms present several challenges requiring resolution.

### 10.3. Trends in the Development and Application of Nucleic Acid Diagnostic Methods for Swine Diseases

In the realm of veterinary medicine, PCR-based diagnostic methods are increasingly being embraced owing to their remarkable sensitivity, exceptional specificity, and rapid turnaround time [240]. Currently, qPCR stands as the most prevalent and widely embraced nucleic acid detection method for diagnosing infectious diseases in pigs. Furthermore, after the ASF epidemic in 2018 and the global COVID-19 pandemic in 2019, there has been a heightened appreciation for the significance of qPCR technology and nucleic acid detection among the populace. Hence, we can foresee that in the forthcoming years, sqPCR and mqPCR assays will continue to be extensively utilized in the diagnosis of porcine diseases. As the COVID-19 pandemic has led to a rapid expansion of qPCR assay production capacity in human medicine, it is anticipated that this capacity will quickly spillover to veterinary medicine as the epidemic subsides. One consequence of this spillover effect is an anticipated further reduction in the price of commercialized qPCR assays used for pig disease diagnosis.

In addition to the conventional qPCR method, which will continue to be utilized for pig disease diagnosis, we anticipate that innovative nucleic acid diagnostic methodologies for pig diseases will persistently progress in three key areas: the NAMT, the POC testing, and the fully integrated and automated all-in-one platform. They may evolve in parallel, each specializing in its own domain of expertise, or they may intermingle and complement one another during their evolution process. Technologies in the NAMT are more likely to be integrated with those in the other two areas to create more powerful and practical novel swine disease diagnostic products. For instance, the integration of multiplex IA technology with LFD to develop POC diagnostic assays for swine diseases represents an innovative approach warranting further comprehensive investigation [23,213].

The NAMT method, with its robust multiplexing capability, would be anticipated to significantly improve acceptance among pig farmers. However, stakeholders should select appropriate NAMT methods based on their unique characteristics to align with specific clinical scenarios, optimizing their utility for diagnosing and preventing porcine diseases. A nucleic acid-based POC diagnostic assay with multiplexing ability, as well as portability, ease of use, and user-friendly features, is particularly suitable for on-site screening of infectious diseases commonly occurring in pig populations, especially in small-scale or backyard pig farms lacking dedicated laboratory facilities. The ideal NAMT method for middle- and large-scale pig farms with modestly equipped diagnostic laboratories is the low- to medium-throughput (the capacity to detect multiple pathogens simultaneously) diagnostic tests, e.g., mqPCR assay and CE-based multiplex assay, that offer reliable, accurate, and straightforward diagnostic results without the need for highly skilled laboratorians and bioinformaticians. These NAMT methods can function as daily surveillance tools in satellite laboratories situated near swine farms. Although microarray and NGS platforms are not yet suitable for routine diagnostic testing, they offer distinct advantages in the analysis of genetic information, mutation characteristics, and evolutionary processes of pathogens. Hence, it is crucial for central laboratories of large-scale swine farms and research institutions to adopt them.

Last but not the least, the integration of NAMT methodology with artificial intelligence (AI) technology and its application in the diagnosis of infectious swine diseases is eagerly anticipated. At present, the research and application of AI in veterinary medicine primarily focus on medical image analysis to assist in animal disease diagnosis and recognizing sound features for monitoring the health status of animal populations [241,242,243]. However, there has been limited exploration and implementation in the sub-areas of pathogen identification, clinical disease prediction, and personalized medicine. This may be attributed to persisting challenges in AI for infectious disease management, including the rapid mutation of pathogens, unpredictable outbreaks of infectious diseases, and issues related to data quality [244]. Fortunately, the integration of genomic, transcriptomic, and other omics data holds great potential to significantly improve the precision of AI in diagnosis and predicting animal infectious disease outbreaks [242], while many technologies in NAMT methods (e.g., NGS and microarray) can make various-omics profiling more accessible in the veterinary field. Consequently, we hold a highly optimistic view regarding the exceptional performance of NAMT combined with AI in pig disease diagnosis. It not only has the potential to address emerging challenges in pig disease diagnosis resulting from the introduction of NAMT, thereby enhancing diagnostic accuracy, but also to forecast the likelihood and trajectory of pig epidemic outbreaks by comprehensively analyzing NAMT diagnostic results, historical outbreak data, environmental hygiene status, as well as weather and seasonal factors. Moreover, it may even propose effective treatment and prevention strategies based on these comprehensive analysis results.

### 10.4. Critical Role of NAMT in Xenotransplantation Biosecurity

The multiplex diagnostic technologies discussed extend beyond conventional veterinary applications to address critical biosecurity challenges in biomedical swine production for xenotransplantation. Pigs represent the primary source of organs for human transplantation owing to their physiological compatibility and scalability; however, the significant risk of cross-species pathogen transmission, such as encompassing porcine endogenous retroviruses (PERVs), porcine cytomegalovirus, and novel uncultivable viruses, poses a major barrier to clinical implementation [245,246]. NAMT provides indispensable solutions for stringent pathogen screening within designated pathogen-free donor pigs. These methods enable comprehensive pathogen surveillance through high-throughput techniques such as microarrays and NGS, which facilitate simultaneous detection of diverse pathogens in single samples, effectively overcoming limitations of cell-culture-based methods for non-cultivable agents. mNGS further enhances surveillance by identifying novel and latent viruses, establishing critical baseline viromes for donor pigs.

Crucially, NAMT offers exceptional sensitivity for low-abundance targets essential for transplant safety. dPCR achieves single-copy sensitivity, enabling detection of PERV integration sites and precise quantification of viral loads in tissues to ensure compliance with safety thresholds. Complementarily, microfluidics-based multiplex assays validate pathogen-free status in pre-transplant organ biopsies while minimizing sample consumption. Furthermore, the integration of NAMT with gene-editing technologies is vital: CRISPR-Cas-modified donor pigs necessitate rigorous validation of edited loci [247], where NGS and mdPCR assays confirm target gene disruption while concurrently screening for potential off-target effects.

The clinical imperative for NAMT is underscored by recent xenotransplantation trials. Reported cases of porcine cytomegalovirus transmission in pig-to-human heart transplants, resulting in thrombocytopenia and graft dysfunction, highlight the necessity for rapid pathogen detection—particularly for agents undetectable by serology [245]. Looking forward, standardizing NAMT panels for xenotransplantation-specific pathogens and implementing POC microfluidic devices for real-time organ screening will accelerate clinical adoption. This application powerfully demonstrates NAMT’s transformative potential in bridging veterinary diagnostics and human medicine, ultimately ensuring the biosecurity of xenotransplantation.

## Data Availability

Data sharing is not applicable to this article as no datasets were generated or analyzed during the current study.
